# CBCT Evaluation of the Relationship Between Mandibular Molars and the Inferior Alveolar Canal in a Peruvian Population

**DOI:** 10.4317/jced.64037

**Published:** 2026-04-25

**Authors:** Yuriani Del Carmen Rebaza-Magán, Marco Antonio Reátegui-Navarro, Cesar Augusto Jiménez-Prado, Manuel Fernando Guillén-Galarza, Hector Martin Vargas-Cornejo

**Affiliations:** 1 DDS (Stomatology). National University of Trujillo. Faculty of Stomatology. Trujillo, Peru; 2 DDS, PhD (Stomatology). National University of Trujillo. Faculty of Stomatology. Trujillo, Peru; 3 DDS, MSc (Stomatology). National University of Trujillo. Faculty of Stomatology. Trujillo, Peru

## Abstract

**Background:**

Proximity between the inferior alveolar canal and mandibular molar apices is a critical factor in oral surgery and implant dentistry, as it may increase the risk of nerve injury. Cone-beam computed tomography (CBCT) enables precise three-dimensional evaluation of this relationship; however, evidence in Peruvian populations remains limited.

**Material and Methods:**

A descriptive cross-sectional study was conducted using 177 CBCT scans obtained from the IRM Radiological Center in Trujillo, Peru (2024-2025). The distance between the inferior alveolar canal and the apices of mandibular first and second molars was measured at the mesial and distal roots. Descriptive statistics were calculated, and differences according to age and sex were assessed using Student's t-test and the Kruskal-Wallis test (p &lt; 0.05).

**Results:**

First mandibular molars showed greater distances from the canal, with mean values of 5.1 mm in mesial roots and 4.3-4.4 mm in distal roots. Second molars presented shorter distances, particularly in distal roots (2.5-2.6 mm). No significant differences were observed among age groups (p &gt; 0.05). Male patients showed greater distances than females, with significant differences in mesial roots (p &lt; 0.05).

**Conclusions:**

Second mandibular molars are located closer to the inferior alveolar canal than first molars. CBCT provides clinically relevant information for surgical planning and may help reduce the risk of inferior alveolar nerve injury.

## Introduction

The anatomical relationship between the inferior alveolar canal (IAC) and the apices of mandibular molars is a key factor in oral surgery and implant dentistry, as inadequate assessment may increase the risk of IAC nerve injury during procedures such as extractions or implant placement. Damage to this structure may result in complications such as lower lip paresthesia, pain, or functional impairment (1). Previous reports indicate that the inferior alveolar nerve is the most frequently injured nerve in dental procedures, accounting for 64.4% of cases, followed by the lingual nerve (28.8%) (2). The inferior alveolar canal is an important mandibular structure that contains the inferior alveolar neurovascular bundle, which comprises the artery, vein, and nerve. Preserving this structure during dental procedures is essential for avoiding irreversible damage (3). The canal begins at the medial surface of the mandibular ramus on the mandibular foramen, extends through the mandibular body, and ends at the mental foramen (4). The canal lies in close proximity to the apices of mandibular premolars and molars due to its anatomical course (5). The mandibular first and second molars are involved in both surgical and endodontic procedures. The first molar usually erupts between six and seven years of age and presents two roots with an average length of approximately 14 mm, whereas the second molar erupts between 11 and 13 years of age and has a similar anatomical structure with shorter roots (6). These teeth represent an important risk area during dental interventions because of their proximity to the inferior alveolar canal (7). Conventional radiographic methods, such as periapical and panoramic radiographs, have been used to evaluate the mandibular canal. However, these techniques have limitations, including image distortion, superimposition of anatomical structures, and inability to perform 3D analysis (8 , 9). Cone-beam computed tomography (CBCT) has become an important imaging modality in dentistry because it provides three-dimensional visualization of craniofacial structures with high spatial resolution and lower radiation doses than conventional CT. This technology allows for the accurate evaluation of the relationship between dental roots and IAC (10). CBCT enables accurate three-dimensional assessment of the relationship between mandibular molar apices and the inferior alveolar canal, facilitating safer surgical planning. Despite its clinical importance, limited quantitative evidence is available for Latin American populations, particularly in Peru. Most studies describe general anatomical patterns without establishing clinically applicable thresholds for risk assessment. Therefore, this study aimed to determine the distance between the inferior alveolar canal and the root apices of mandibular first and second molars using CBCT, provide population-specific reference values, and identify clinically relevant proximity thresholds to improve surgical planning and reduce the risk of inferior alveolar nerve injury.

## Material and Methods

This retrospective study analyzed CBCT images obtained between 2024 and 2025 from the digital database of the IRM Radiological Center in Trujillo, Peru, between 2024 and 2025. All tomographic records were anonymized before analysis to ensure patient confidentiality. A designated researcher assigned a unique identification code to each CBCT scan independent of the examiner, thereby concealing personal information such as patient names and identification numbers. The images were subsequently evaluated to determine the distance between the inferior alveolar canal and the root apices of the mandibular first and second molars. - Sample Selection A previous CBCT study reported a mean distance of approximately 6.06 mm between the inferior alveolar canal and the mandibular molar apices (11). The sample size was calculated using the following formula for estimating a mean in a finite population: n = (N × Z² × ²) / [(N 1) × E² + Z² × ²]. Where n is the required sample size, N is the population size, Z is the standard normal value corresponding to the desired confidence level, is the standard deviation, and E is the margin of error. The calculation was based on a population of N = 400 cone-beam CT scans available in the IRM Radiological Center database. A 95% confidence level was assumed (Z = 1.96), with a margin of error of 0.5 mm (E = 0.5) and a reference standard deviation of 2.27 mm ( = 2.27), derived from previous CBCT studies evaluating the distance between the inferior alveolar canal and mandibular molar apices. These parameters ensured adequate precision in the estimation of mean distance. Based on this calculation, the required sample size was 177 CBCT scans. The sample was selected from the IRM Radiological Center database (2024-2025) using simple random sampling to ensure that all records meeting the inclusion and exclusion criteria had an equal selection probability. - Inclusion Criteria The CBCT scans of patients aged 18-60 years were included in the analysis. The images were required to show fully erupted mandibular first and second molars with complete root development, as well as adequate image quality and resolution to allow clear identification of the inferior alveolar canal and root apices. Although the inclusion criteria included patients aged 18-60 years, individuals older than 45 years were excluded from subgroup analysis due to insufficient sample size, but were retained in the overall sample. - Exclusion Criteria CBCT scans presenting pathological lesions in the posterior mandibular region or evidence of previous surgical interventions in the mandibular molar area were excluded. Teeth with root resorption, fractures, or severe anatomical alterations were also excluded. Additionally, images with artifacts or insufficient quality that prevented accurate measurement were excluded from the analysis. - Data Collection CBCT scans were obtained using a Carestream CS 9300 unit with a voxel size of 0.2 mm and a field of view of 10 × 10 cm. Each image was evaluated in the sagittal and coronal planes to identify the inferior alveolar canal and the root apices of the mandibular first and second molars. Linear measurements were obtained as the shortest distance between the root apex and the inferior alveolar canal for each molar root. All measurements were recorded in millimeters using the software integrated into the CBCT system and registered in a data collection sheet designed for the study. To ensure measurement reliability, the examiner underwent a calibration process prior to data collection. A subset of images was randomly selected and re-evaluated after a two-week interval. Intra-observer agreement was assessed using the intraclass correlation coefficient (ICC), which demonstrated excellent reproducibility (ICC = 0.92). - Statistical Analysis The collected data were analyzed using IBM SPSS Statistics software (version 27.0; IBM Corp., Armonk, NY, USA). Descriptive statistics, including mean, median, standard deviation, and interquartile range, were calculated. For inferential analysis, Student's t-test or the Kruskal-Wallis test was used according to the data distribution. Statistical significance was set at p &lt; 0.05.

## Results

A total of 177 CBCT scans were included. Females represented 71.8% of the sample, while males accounted for 28.2%. Regarding age distribution, the 18-26-year group was the most represented (46.9%). The results showed variations in the distance between the inferior alveolar canal and the dental roots depending on the molar and the root analyzed. In mandibular first molars (teeth 3.6 and 4.6), greater distances were observed in both mesial and distal roots. The mean distance in mesial roots was approximately 5.1 ± 2.0 mm, while distal roots showed mean values of 4.4 ± 2.1 mm (tooth 3.6) and 4.3 ± 2.0 mm (tooth 4.6). These findings indicate that the root apices of first molars tend to be located farther from the inferior alveolar canal. In contrast, second mandibular molars (teeth 3.7 and 4.7) presented shorter distances, particularly in distal roots, with mean values of 2.6 ± 1.9 mm and 2.5 ± 1.7 mm, respectively. Mesial roots also showed reduced distances, ranging from 2.1 to 3.2 mm (Table 1).

Table 1

When analyzed by age groups (18-26, 27-36, and 37-45 years), mean and median values remained relatively stable. In teeth 3.6 and 4.6, mesial root values were close to 5 mm, with moderate variability (standard deviation: 1.8-2.2 mm). In teeth 3.7 and 4.7, a slight increase in mean distance with age was observed; however, these differences were not statistically significant (p &gt; 0.05). A similar pattern was observed in distal roots, where first molars remained stable (4.0-4.6 mm), while second molars showed a slight increase with age, particularly in tooth 3.7 (2.4 to 3.3 mm). Nevertheless, these differences were not statistically significant (H = 1.49-5.57; p = 0.062-0.475) (Table 2).

Table 2

Regarding sex differences, males showed greater distances than females. Significant differences (p &lt; 0.05) were observed in the mesial roots of teeth 3.6, 4.6, 3.7, and 4.7. In distal roots, statistical significance was found only in first molars (3.6 and 4.6), whereas differences in second molars (3.7 and 4.7) were not significant (p &gt; 0.05). Data dispersion, reflected by standard deviation, was similar between groups (Table 3).

Table 3

## Discussion

The present study evaluated the anatomical relationship between the inferior alveolar canal and the root apices of mandibular first and second molars using CBCT. The findings confirm that second mandibular molars are located closer to the inferior alveolar canal than first molars, consistent with previously reported anatomical patterns. Importantly, this study provides population-specific quantitative reference values that may improve clinical risk assessment in Peruvian patients. The mean distances observed for second molars (2.6 ± 1.9 mm for tooth 3.7 and 2.5 ± 1.7 mm for tooth 4.7) are consistent with those reported by Díaz et al. (12), who found an average distance of 2.82 mm. Similarly, the mean distance of 5.1 mm for the mesial root of first molars aligns with the range reported by Sharaan et al. (13) (5.05-5.52 mm), although slightly lower values have been described by Aqili et al. (14) (4.79 ± 2.29 mm), suggesting population-related anatomical variability. From a clinical perspective, distances below 3 mm between the root apex and the inferior alveolar canal may represent a clinically relevant threshold associated with an increased risk of nerve injury during surgical and endodontic procedures. In this study, distal roots of second mandibular molars frequently showed distances below this threshold, indicating close anatomical proximity to the inferior alveolar canal. These findings support the use of CBCT as a preoperative tool in cases with suspected proximity, allowing for more accurate risk assessment and safer treatment planning (15). No statistically significant differences were observed across age groups, with relatively stable measurements within the evaluated range. These results are consistent with those of Razumova et al. (11), who reported no significant age-related differences despite a tendency toward shorter distances in younger individuals. Within the studied age range (18-45 years), age appears to have limited influence on this anatomical relationship. Sex-based analysis revealed greater distances in male patients, with significant differences in mesial roots (p &lt; 0.05). Similar findings have been reported by Vidya et al. (16), suggesting that sex-related anatomical differences may be relevant during surgical planning in the mandibular posterior region. Previous CBCT studies have highlighted anatomical variability in the relationship between mandibular molar roots and the inferior alveolar canal, reinforcing the importance of individualized imaging assessment to reduce the risk of nerve injury (17 , 18). This study has several strengths, including the use of high-resolution CBCT imaging, standardized measurement protocols, and random sampling, which enhance the reliability and reproducibility of the findings. However, some limitations should be considered. The study was conducted in a single radiological center, which may limit external validity. Additionally, the predominance of female participants may have influenced sex-based comparisons. As a cross-sectional study, it was not possible to evaluate longitudinal changes in this anatomical relationship. Future multicenter studies are recommended to improve generalizability. Despite these limitations, this study provides clinically applicable reference values and identifies proximity thresholds that may contribute to safer surgical planning in underrepresented populations.

## Conclusions

Second mandibular molars are located closer to the inferior alveolar canal than first molars. Distances below 3 mm may represent a clinically relevant threshold for increased risk of nerve injury. CBCT is recommended for accurate preoperative evaluation to improve clinical decision-making and reduce complications.

## Figures and Tables

**Table 1:** Distance between the inferior alveolar canal and root apices of mandibular first and second molars measured by CBCT (mm).

		Ápex	
Molar	Pza	Mesial root	Distal root
		Mean ± SD	Mean ± SD
1er molar	3.6	5.1 ± 2.0	4.4 ± 2.1
	4.6	5.1 ± 2.0	4.3 ± 2.0
2do molar	3.7	3.2 ± 2.1	2.6 ± 1.9
	4.7	3.2 ± 2.0	2.5 ± 1.7

1

**Table 2:** Distance between the inferior alveolar canal and molar root apices according to age groups.

	Age (years)			test
Root, piece, and indicator	18 - 26	27 - 36	37 - 45	
Mesial root				
Pza 3.6				
Mean	5.0	5.1	5.1	H = 0.08
Median	4.7	4.6	5.1	
Standard deviation	1.9	2.2	2.1	p = 0.959
IQR	3.0	3.5	2.5	
Pza 4.6				
Mean	4.9	5.3	4.9	H = 0.86
Median	5.0	5.1	4.7	
Standard deviation	2.0	2.1	1.8	p = 0.652
IQR	2.6	3.5	2.9	
Pza 3.7				
Mean	2.9	3.4	3.9	H = 1.49
Median	2.3	3.1	3.4	
Standard deviation	2.0	2.2	2.1	p = 0.475
IQR	2.9	3.1	2.8	
Pza 4.7				
Mean	2.8	3.6	3.4	H = 3.68
Median	2.4	3.5	3.3	
Standard deviation	1.9	2.1	1.6	p = 0.159
IQR	2.8	3.4	1.9	
Distal root				
Pza 3.6				
Mean	4.2	4.5	4.5	H = 3.01
Median	3.9	3.9	4.5	
Standard deviation	2.0	2.2	1.8	p = 0.159
IQR	3.1	3.3	2.2	
Pza 4.6				
Mean	4.0	4.6	4.3	H = 3.85
Median	3.5	4.3	4.4	
Standard deviation	2.0	2.1	1.5	p = 0.146
IQR	2.5	3.2	2.5	
Pza 3.7				
Mean	2.4	2.8	3.3	H = 5.57
Median	1.8	2.5	2.8	
Standard deviation	1.8	1.9	1.9	p = 0.062
IQR	3.0	2.7	3.1	
Pza 4.7				
Mean	2.2	2.8	2.6	H = 3.54
Median	2.1	2.4	2.1	
Standard deviation	1.7	1.9	1.5	p = 0.170
IQR	1.9	3.3	2.2	

*Kruskal-Wallis H statistical test

**Table 3:** Comparison of the distance between the inferior alveolar canal and molar root apices according to sex.

	Sex		test
Root, piece, and indicator	Male	Female	
Mesial root			
Pza 3.6			
Mean	5.5	4.9	H = 4.01
Median	5.4	4.4	
Standard deviation	1.9	2.0	p = 0.045
IQR	3.0	3.1	
Pza 4.6			
Mean	5.7	4.9	H = 4.33
Median	5.7	4.6	
Standard deviation	1.9	2.0	p = 0.038
IQR	2.9	2.7	
Pza 3.7			
Mean	3.8	3.0	H = 6.60
Median	3.3	2.7	
Standard deviation	2.2	2.0	p = 0.010
IQR	3.4	2.8	
Pza 4.7			
Mean	3.5	3.0	H = 5.45
Median	3.1	2.8	
Standard deviation	1.9	2.0	p = 0.020
IQR	3.0	3.1	
Distal root			
Pza 3.6			
Mean	4.8	4.2	H = 4.62
Median	4.9	3.9	
Standard deviation	1.9	2.1	p = 0.032
IQR	2.9	3.1	
Pza 4.6			
Mean	4.8	4.1	H = 4.01
Median	4.4	3.9	
Standard deviation	1.9	2.1	p = 0.045
IQR	3.1	2.6	
Pza 3.7			
Mean	3.0	2.4	H = 2.78
Median	2.8	2.0	
Standard deviation	1.9	1.9	p = 0.095
IQR	2.7	2.9	
Pza 4.7			
Mean	2.7	2.4	H = 1.30
Median	2.4	2.0	
Standard deviation	1.7	1.8	p = 0.254
IQR	2.9	2.3	

*Kruskal-Wallis H statistical test

## Data Availability

Informed Consent StatementPatient consent was waived due to the retrospective design of the study and the use of anonymized CBCT images obtained from an existing radiological database.
